# The last bite on the plate: association of plate-clearing tendency and sustainable nutrition with weight gain in pregnancy

**DOI:** 10.1017/S0007114526107387

**Published:** 2026-05-28

**Authors:** Ceren Şarahman-Kahraman, Cansu Memiç-İnan, Açelya Hür

**Affiliations:** 1 Department of Nutrition and Dietetics, University of Alanya Alaaddin Keykubat, Alanya/Antalya, Türkiye; 2 Department of Nutrition and Dietetics, University of Hitit, Çorum, Türkiye; 3 Alanya Alaaddin Keykubat University Alanya Education and Research Hospital, Gynaecology and Obstetrics, Alanya/Antalya, Türkiye

**Keywords:** Eating behaviors, Gestational weight gain, Nutrition, Pregnancy

## Abstract

The aim is to examine the relationship between factors thought to potentially influence weight gain, such as sustainable nutrition (SN) behaviour and plate-clearing tendency (PCT) during pregnancy and gestational weight gain (GWG). This cross-sectional correlational study was conducted on 340 women in the last trimester of pregnancy. Study data were collected through face-to-face interviews using a questionnaire form between October and December 2024. PCT is lower among younger women, those with low income and those with insufficient GWG (*P* < 0·05). SN behaviours are higher in those who are older, have higher education levels, have lower income and have moderate physical activity (*P* < 0·05). A negative correlation was found between food preference, a component of SN behaviours, and GWG (*P* < 0·05). In the binary logistic regression model, higher pre-pregnancy BMI significantly increased the likelihood of excessive GWG (OR = 1·49, 95 % CI 1·332, 1·665, *P* < 0·001), whereas high physical activity was found to be protective against excessive GWG (OR = 0·214, 95 % CI 0·061, 0·747, *P* = 0·016). It was determined that pre-pregnancy BMI was higher and physical activity was lower in those with excessive GWG; in addition, food preference, one of the factors of SN behaviour, affected weight gain. Food preference can be considered as a factor that may affect GWG.

Sustainable nutrition (SN) enables a diet that is accessible, environmentally friendly and health-promoting for both present and future generations, while protecting natural food sources and supporting ecosystem health^(1)^. Because SN is an approach that both supports individual health and prioritises the conservation of environmental resources^(2)^, the WHO and the FAO have also emphasised that healthy diets should support sustainable food systems and have published the guide ‘Sustainable Healthy Diets – Guiding Princip’es’^(3)^. During pregnancy, nutrition plays a critical role in maternal and fetal health, and there is growing recognition that sustainable dietary behaviours may also be relevant in this period^(3,4)^.

Nutrition during pregnancy is important in terms of protecting maternal health and supporting the healthy development of the fetus in both the short and long term. Today, it is known that the environmental, economic and social dimensions of nutrition are also taken into consideration rather than focusing on the effect of dietary habits on individual health and the consumption of certain nutrients during pregnancy^(5)^. In addition, it is stated that pregnancy period may be a process involving more motivation in terms of improving dietary habits, increasing orientation towards healthy food preferences and providing positive lifestyle changes^(6)^. In this process, the adoption of sustainable dietary behaviour and high adherence to a sustainable dietary pattern such as the Mediterranean diet not only provides environmental benefits but also promotes individual health through improved nutritional quality and reduced nutritional deficiencies^(7,8)^. However, there is also concern that the consumption of environmentally sustainable foods may increase the risk of nutrient deficiencies during pregnancy^(9)^. In a study, the high-emission food most frequently consumed by 171 pregnant women (beef) was replaced with sustainable alternatives (tofu and legumes), significantly reducing environmental impact without causing nutrient deficiencies^(9)^. In this context, it is important for individuals to be aware of behaviours that negatively impact sustainability and to implement behavioural changes.

SN not only refers to a shift towards eating habits with less harmful effects on the environment but also aims to reduce overconsumption and food waste^(10)^. Therefore, plate-clearing tendency (PCT) is a concept that may affect SN. The PCT, which refers to the approach to finish all the food on the plate while eating, is a learned behaviour that includes morals (avoiding food waste, environmental responsibility and respect for food as valuable) and economic concerns about waste, and this tendency can lead to overconsumption, leading to an increase in total energy intake and thus body weight^(11)^. Considering the high increase in appetite in the last trimester of pregnancy, if foods with high energy content are offered in large portions, women may exhibit overeating behaviour by ignoring satiety signals^(12)^. Frequent observation of the PCT during pregnancy and making this situation a habit may lead to excessive gestational weight gain (GWG) and may lead to the emergence of unwanted health problems during pregnancy^(13)^.

Pregnancy is a process of weight gain by nature, and it is reported that approximately 47 % of pregnant women worldwide have GWG above the recommended^(14)^. While insufficient GWG increases the risk of preterm and low birth, excessive GWG leads to pregnancy-induced hypertension, hyperglycaemia and macrosomia and increases the risk of obesity development in both mother and infant in the postnatal period^(15,16)^. Maternal diet and feeding behaviours during pregnancy are extremely important in achieving optimal GWG. A study conducted among pregnant women in Michigan reported that 43·7 % of the participants experienced excessive GWG, and that higher consumption of vegetables and fruits could help reduce excessive GWG in obese women^(17)^. Therefore, the hypothesis of our study is that low PCT and adherence to sustainable eating habits may be associated with healthy GWG in pregnant women. The dependent variable of the study is GWG, while the independent variables are PCT and SN. Considering the importance of GWG in terms of maternal and infant health, there are few, if any, studies that have directly investigated SN and PCT in relation to GWG. Therefore, the aim of this study was to investigate the relationship between SN and PCT with GWG in women aged 18–45 years in the last trimester of pregnancy, who were followed at the Gynecology and Obstetrics Outpatient Clinic of Alanya Alaaddin Keykubat University Training and Research Hospital between October and December 2024.

## Materials and methods

### Study design and subject recruitments

The sample of this cross-sectional study consisted of women aged 18–45 years who were in the last trimester of pregnancy (28–40/41 weeks) and were followed up in the Gynaecology and Obstetrics Outpatient Clinic of Alanya Alaaddin Keykubat University, Training and Research Hospital. Participants were selected using a consecutive sampling method from among pregnant women who met the inclusion criteria during the defined study period. During the defined study period (October–December 2024), approximately 420 pregnant women were invited to participate in the survey, and 340 agreed to take part in the study; accordingly, the participation rate was determined to be approximately 80·9 %. Within the scope of the study, 340 women aged 19–44 years, literate, in the last trimester of pregnancy (28–40/41 weeks) and willing to participate in the study constituted the sample. Those with conditions such as multiple pregnancy, gestational diabetes or pre-eclampsia, those on disease-specific diets and those with a history of eating disorders were excluded from the study.

### Sample size calculation

The sample size was calculated using G*Power software (version 3.1). Based on the effect size reported in the study by Tebbani *et al*.^(18)^, with an *α* level of 0·05 and statistical power of 90 %, the minimum required sample size was estimated to be 280 pregnant women.

### Study questionnaire

The questionnaire form consists of multiple sections designed to collect comprehensive data from participants; the first section includes multiple-choice questions on demographic information, the second and third sections comprise previously validated scales (Behaviors Scale Towards Sustainable Nutrition and Plate Clearing Tendency Scale) and the fourth section contains anthropometric measurements (pre-pregnancy body weight based on self-report, current body weight and height). The study data were collected through face-to-face interviews using a questionnaire form.

In the general information section of the questionnaire, in addition to multiple-choice questions prepared by the researchers to determine participants’ age, education level, occupation and income, questions aimed at assessing eating habits, including the number of main and intermediate meals, meal skipping and snack consumption during pregnancy, were collected. The WHO recommends at least 150 min of moderate physical activity (brisk walking, swimming, etc.) per week during pregnancy^(19)^. For this reason, in order to determine the physical activity level of pregnant women, the question ‘how many minutes of moderate physical activity do you do at least per week’ was asked and those who did less than 150 min of physical activity were considered low, those who did 150 min were considered moderate and those who did more than 150 min were considered high.

#### Behaviours scale towards sustainable nutrition

In order to evaluate the SN behaviours of individuals, Garipoglu *et al*.^(20)^ developed the Behaviours Scale Towards Sustainable Nutrition. This scale, which is scored on a five-point Likert scale from 1 = never to 5 = always, consists of twenty-nine items and four dimensions: food preference, food waste reduction, seasonal and local food consumption, and food purchase. There are items 1, 2, 3, 4, 5 and 6 in the food preference dimension, items 7, 8, 9, 10, 11, 12, 13, 14 and 15 in the food waste reduction dimension, items 16, 17, 18, 19, 20, 21, 22 and 23 in the seasonal and local food consumption dimension and items 24, 25, 26, 27, 28 and 29 in the food purchase dimension. There are no reverse-coded items in the scale. The lowest score that can be obtained from the scale is 29, the highest score is 145 and the Cronbach *α* value of the scale is 0·92. Sub-dimension scores are obtained by dividing the sum of the scores given by the individuals to the questions in the sub-dimension by the number of questions in that sub-dimension. The higher total score and sub-dimension scores indicate that the individual has more SN behaviours. In this study, Cronbach’s *α* value of the scale was found to be 0·940.

#### Plate Clearing Tendency Scale

In order to measure individuals’ tendency to finish the food on their plates, ‘the Plate Clearing Tendency Scale’ developed by Robinson *et al*.^(11)^ and validated in Turkish by Şarahman Kahraman *et al*.^(21)^ was used. This scale, which is a five-point Likert scale ranging from 1 = strongly disagree to 5 = strongly agree, consists of five items in total and there are no reverse-coded items. The Cronbach *α* value of the Turkish version of the scale is 0·70, and the McDonald Omega value is 0·71. The total score is obtained by summing the scores for the five items. Higher scores on the scale indicate that this tendency (i.e. the tendency to finish all the food on the plate) is stronger. In this study, Cronbach’s *α* value of the scale was found to be 0·593. For reliability measurements, values below 0·50 are considered low reliability, values between 0·50 and 0·80 are considered moderately reliable and values above 0·80 are considered highly reliable^(22)^.

#### Anthropometric measurements

The pre-pregnancy body weight of women in the last trimester of pregnancy included in the study was based on self-report. Body weight (kg) at the time of participation in the study was measured using a calibrated precision scale sensitive to 0·1 kg, and height (cm) was measured using a stadiometer in the Frankfort [horizontal] plan^(23)^. Pre-pregnancy BMI was calculated by dividing pre-pregnancy body weight by the square of height squared (kg/m^2^). According to the WHO’s BMI classification for adults, < 18·5 kg/m^2^ is underweight, 18·5–24·9 kg/m^2^ is normal, 25·0–29·9 kg/m^2^ is overweight and ≥ 30·0 kg/m^2^ is obese^(24)^. GWG was evaluated according to the Institute of Medicine (IOM) recommendations; total GWG of 11·5–16·0 kg in women with a BMI of 18·5–24·9 kg/m^2^, 7·0–11·5 kg in women with a BMI of 25·0–29·9 kg/m^2^ and 5·0–9·0 kg in women with a BMI ≥ 30·0 kg/m^2^ were categorised as normal, below these references as insufficient and above these references as excessive GWG^(25)^.

### Statistical analysis

IBM SPSS Statistics (version 26) software was used to analyse the data obtained from this study. Normality assumption was assessed both analytically (hypothesis tests) and visually (histograms, Q–Q graphs and box plots). Descriptive data were presented with number-percentage tables. Normally distributed variables were presented using mean and standard deviation, and non-normally distributed variables were presented using median, minimum and maximum values. Parametric tests were preferred for normally distributed data, and non-parametric tests were preferred for non-normally distributed data. Independent-sample *t* test was applied for pairwise group comparisons, and one-way ANOVA or Kruskal–Wallis test was applied for three or more group comparisons. The correlation between two quantitative variables was determined by Pearson’s correlation coefficient. Binary logistic regression analysis was applied to determine the factors predicting excessive weight gain during pregnancy. Significance level was accepted as *P* < 0·05 in all analyses.

## Results

Table 1 provides general information about the participants. The mean age of participants was 30·0 (sd 7·3) years, 50·6 % were between 31 and 44 years old, 37·1 % were primary/secondary school graduates and 38·8 % were high school graduates. 69·7 % of the participants have an income above the minimum wage. The mean pre-pregnancy BMI was 24·1 (sd 3·1) kg/m^2^, and 57·4 % of the participants had normal body weight, 37·9 % were overweight, 2·9 % were underweight and 1·8 % were obese. It was determined that 34·4 % of the participants had excessive and 22·4 % had insufficient GWG. The physical activity levels of the participants were mostly moderate (46·2 %) and low (45·6 %). The mean PCT score was 17·9 (sd 1·9), and the SN score was 65·4 (sd 15·8). 14·4 % of the participants reported skipping meals regularly, and 65·3 % reported skipping meals sometimes. The most frequently skipped meal was snacks (50·6 %), followed by breakfast (19·4 %) and lunch (8·2 %). Yogurt, milk, fruit (18·8 %) and nuts (18·7 %) were the most preferred snacks, while salad was the least consumed food (2·4 %).

**Table 1.** General characteristics of participants Table 1 long description.

		*n*	%	Mean	sd
Age (years)				30·0	7·3
Age groups	19–30	168	49·4		
	31–44	172	50·6		
Education status	Primary/secondary school	126	37·1		
	High school	132	38·8		
	Bachelor’s and postgraduate	82	24·1		
Income	Below the minimum wage	29	8·5		
	Minimum wage	74	21·8		
	Above the minimum wage	237	69·7		
Pre-pregnancy BMI (kg/m^2^)				24·1	3·1
Pre-pregnancy BMI, classification	Underweight (< 18·5 kg/m^2^)	10	2·9		
	Normal (18·5–24·99 kg/m^2^)	195	57·4		
	Overweight (25·0–29·99 kg/m^2^)	129	37·9		
	Obesity (≥ 30 kg/m^2^)	6	1·8		
Gestational weight gain classification	Insufficient weight gain	76	22·4		
	Normal weight gain	147	43·2		
	Excessive weight gain	117	34·4		
Physical activity level	Low	155	45·6		
	Moderate	157	46·2		
	High	28	8·2		
Plate-clearing tendency score				17·9	1·9
Sustainable nutrition score				65·4	15·8
*Eating habits*					
Meal skipping behaviour	Yes	49	14·4		
	Sometimes	222	65·3		
	No	69	20·3		
Skipped meal	Breakfast	66	19·4		
	Lunch	28	8·2		
	Dinner	5	1·5		
	Snack meals	172	50·6		
Foods consumed during snacks^*^	Biscuit, cracker	230	16·3		
	Bagel, cake and pastries	246	17·4		
	Sugar, chocolate, sweets	179	12·7		
	Nuts	264	18·7		
	Yogurt, milk, fruit	266	18·8		
	Salad	34	2·4		
	Sugary and carbonated drinks	193	13·7		

* Participants could select multiple options; percentages are of total snack choices reported.

Information on PCT and SN scores according to the study variables is given in Table 2. It was found that the PCT score showed a significant difference according to age, income and GWG classification (*P* < 0·05). Additionally, the PCT was lower in those aged 19–30 years, those with an income below the minimum wage and those with insufficient GWG (*P* < 0·05). It was determined that the SN score differed significantly according to age, education level, income, physical activity level and meal skipping status (*P* < 0·05). SN score was found to be significantly higher in participants aged 31–44 years, having bachelor’s and postgraduate education level, income below minimum wage, moderate physical activity and skipping meals (*P* < 0·05).

**Table 2.** Plate-clearing tendency and sustainable nutrition scores according to study variables Table 2 long description.

	Plate-clearing tendency score			Sustainable nutrition score		
	Mean	sd	*P*	Mean	sd	*P*
Age						
19–30 years	17·6	2·0	0·001*	63·3	16·1	0·018*
31–44 years	18·3	1·7		67·4	15·2	
Education status						
Primary/secondary school	17·9	1·9	0·879	64·8	12·5^a^	0·002*
High school	17·9	1·7		62·8	15·5^a^	
Bachelor’s and postgraduate	18·0	2·0		70·5	19·2^b^	
Income				Median	Minimum–Maximum	
Below the minimum wage	18·9	1·5^a^	< 0·001*	86·0	39·0–124·0^a^	< 0·001*
Minimum wage	18·4	1·6^a^		67·5	44·0–95·0^b^	
Above the minimum wage	17·7	1·9^b^		62·0	29·0–105·0^b^	
Pre-pregnancy BMI				Mean	sd	
< 25 kg/m^2^	17·8	2·0	0·121	64·6	15·9	0·284
≥ 25 kg/m^2^	18·1	1·6		66·5	15·5	
Gestational weight gain classification						
Insufficient weight gain	17·4	2·2^a^	0·036*	64·6	16·7	0·748
Normal weight gain	18·1	2·0^b^		65·0	15·0	
Excessive weight gain	18·1	1·6^b^		66·2	16·1	
Physical activity level						
Low	17·8	2·2	0·192	65·5	14·9^a^	< 0·001*
Moderate	18·0	1·5		83·2	18·3^b,^^†^	
High	18·4	2·1		62·0	13·9^a^	
Meal skipping behaviour						
Yes	17·5	2·5	0·169	72·5	16·4^a^	0·001*
No	18·0	1·8		64·9	16·2^b^	
Sometimes	18·0	2·1		61·8	11·9^b^	

Independent-samples *t* test was used for normally distributed two-group data, ANOVA for three-group data and Kruskal–Wallis test for non-normally distributed data.

*P < 0·05.

Different superscript letters within a column and variable group indicate significant differences (*P* < 0·05) based on *post hoc* tests.

† Value verified; no data entry error.

Table 3 shows the correlation of SN and its sub-dimensions and PCT score with the study variables. There were significant positive relationships between the SN score and its sub-dimensions of food preference (*r* = 0·843, *P* < 0·001), food waste reduction (*r* = 0·890, *P* < 0·001), seasonal and local food consumption (*r* = 0·916, *P* < 0·001) and food purchase (*r* = 0·747, *P* < 0·001). In addition, a significant positive correlation was found between SN score and PCT score (*r* = 0·136, *P* < 0·05). It was determined that the PCT score was positively associated with seasonal and local food consumption (*r* = 0·186, *P* < 0·001) and food purchase (*r* = 0·140, *P* < 0·001) among the sub-dimensions of the SN scale. GWG was negatively associated with food preference (*r* = –0·115, *P* < 0·05).

**Table 3.** Correlation of sustainable nutrition and its sub-dimensions, and plate-clearing tendency scores with study variables Table 3 long description.

	Sustainable nutrition score	Food preference	Food waste reduction	Seasonal and local food consumption	Food purchase	Plate-clearing tendency score	Age	Pre-pregnancy BMI	Gestational weight gain
Sustainable nutrition score	1	0·843**	0·890**	0·916**	0·747**	0·136*	0·086	0·006	0·003
Food preference		1	0·613**	0·781**	0·463**	0·098	−0·027	−0·101	−0·115*
Food waste reduction			1	0·737**	0·607**	0·062	0·133*	0·004	0·052
Seasonal and local food consumption				1	0·592**	0·186**	0·063	0·009	0·013
Food purchase					1	0·140**	0·122*	0·142**	0·063
Plate-clearing tendency score						1	0·115*	0·050	0·066
Age							1	0·196**	0·049
Pre-pregnancy BMI								1	0·080
Gestational weight gain									1

The Pearson correlation was used.

*P < 0·05, **P < 0·001.

The results of the binary logistic regression model in Table 4 show the factors predicting excessive GWG. It was found that as pre-pregnancy BMI increased, the likelihood of excessive GWG increased significantly (OR = 1·490, %95 CI 1·332, 1·665, *P* < 0·001). High levels of physical activity significantly reduce the likelihood of excessive GWG (OR = 0·214, %95 CI 0·061, 0·747, *P* = 0·016). PCT score, SN score, age, education level and income did not show a significant association with excessive GWG (*P* > 0·05).

**Table 4.** Factors predicting the likelihood of excessive gestational weight gain (as predicted by the binary logistic regression model) Table 4 long description.

Variable	OR	95 % CI for OR	*P*
		Lower, upper	
Plate-clearing tendency score	1·053	0·900, 1·232	0·518
Sustainable nutrition score	1·009	0·989, 1·029	0·386
Pre-pregnancy BMI	1·490	1·332, 1·665	<0·001*
Age	1·006	0·966, 1·047	0·778
Education level (bachelor’s and postgraduate)	1·659	0·803, 3·428	0·172
Income (above minimum wage)	0·575	0·189, 1·747	0·329
Physical activity level (high)	0·214	0·061, 0·747	0·016*

The binary logistic regression analysis was used.

*P < 0·05.

## Discussion

In this cross-sectional study, it was aimed to determine the relationship between SN behaviour and PCT with GWG. The assessment of the potential relationships of these variables with GWG constitutes the original value of the study. However, as a result of the analyses, it was found that PCT and SN behaviour did not show a significant relationship with GWG. Our regression analyses showed that an increase in pre-pregnancy BMI increased the likelihood of excessive GWG, whereas a high level of physical activity significantly decreased this likelihood.

In this study, 65·3 % of the pregnant women who participated in this study stated that they sometimes skipped meals, and it was observed that the most skipped meal was snacks. Although the majority of snackers (19 %) consume healthy options such as milk, yogurt and fruit, snacks with high carbohydrate and sugar content such as biscuits and crackers (16 %), bagels, cakes and pastries (17 %) and sugary and carbonated drinks (14 %) are also consumed. In a study, it was determined that pregnant women had higher consumption of fruits, dairy products and whole grains compared with other women (with or without children)^(26)^. In a study conducted in Poland, it was observed that pregnant women’s consumption of sweets and white bread was high, fish and milk consumption was insufficient, and the nutritional behaviours of pregnant women with higher education level were better^(27)^. It has been reported that most women with low levels of education and income may not pay attention to nutritional recommendations during pregnancy and may encounter inadequacy in macro- and micronutrient intake^(28)^. In a study examining diet and diet during pregnancy, it was determined that those with low education and income levels were fed less (inadequate) and had poor eating habits^(29,30)^. Among the pregnant women who participated in this study, 75·9 % had a low level of education (high school and primary education) and 30·3 % had an income of minimum wage or less. Consistent with the literature, having a low level of education and income may negatively affect the dietary habits of pregnant women. In this direction, it may be useful to create targeted nutrition education programmes for low socio-economic groups.

While 57·4 % of the women participating in this study had normal BMI before pregnancy, 37·9 % were overweight. It was found that 34·4 % of the pregnant women had excessive GWG. In the last 50 years, economic developments, urbanisation and technological advances in food processing have affected both the availability of food and portion sizes at meals^(31)^. Pregnant women are recommended to pay attention to portion sizes to ensure weight control without energy restriction^(32)^. The tendency to completely consume the food on the plate without portion control, that is, the PCT, can be considered as a behaviour that may be associated with GWG^(33)^. In some studies, the PCT was found to be higher in overweight individuals compared with those with normal weight, and PCT has been associated with an increase in BMI^(11,34)^. In the present study, pregnant women with normal and high GWG had a higher PCT compared with those with low GWG. Although a weak positive association was found between the PCT and pre-pregnancy BMI or GWG, this association was not statistically significant. These results suggest that the effect of PCT on GWG may be limited.

The effects of people’s dietary behaviour on both their own health status and the environment are too important to be ignored^(35)^. It is known that SN is associated with the protection of health and environmental resources for future generations and is influenced by various factors^(2)^. One of the important factors for the development of environmental awareness and the increase in the orientation towards SN is the level of education^(36)^. It is stated that young individuals with a high level of education may have high environmental awareness and may make more conscious choices towards SN^(36)^. However, the relationship between SN and age is contradictory, and it should not be ignored that the tendency towards Western-type diets in young people today may constitute an obstacle to sustainable eating behaviour^(37)^. In our study, it was found that SN scores were higher in pregnant women with higher age and education level. It is an expected result that older pregnant women may prefer more traditional dietary methods and prefer environmentally friendly food preferences with increasing education level. Sustainable eating behaviour can also be influenced by the economic situation. One study found that healthy and sustainable dietary patterns are 25–29 % more cost-effective in low-income and lower-middle-income countries^(38)^. In another study, it was determined that sustainable eating behaviours were more common in younger women and those with higher income levels^(39)^. Although education and income level are considered to be intertwined concepts, they have some differences. In a way, individuals with higher levels of education may have higher awareness of sustainability, but on the other hand, individuals with higher levels of education and income may exhibit higher energy and unsustainable eating behaviours as they have more economic freedom^(40)^. Therefore, it is necessary to evaluate the two concepts comprehensively in terms of sustainability. In our study, SN behaviour was found to be high in pregnant women with low income level. This may be thought to be due to the fact that pregnant women avoid wasting food due to low income and tendency to finish the food on their plates.

This study found that pregnant women who skipped meals had higher SN scores, which may be considered an unexpected result, as meal skipping is generally an undesirable behaviour during pregnancy. However, SN is not influenced solely by the number of meals; it may be associated with many factors such as food choices, conscious consumption and food waste. Because any food consumed beyond an individual’s energy requirements leads to increased greenhouse gas emissions, depletion of natural resources and loss of biodiversity, thereby negatively affecting sustainability^(41)^. Additionally, it is stated that meal skipping may support sustainability by leading to reduced food, packaging and waste production through decreased energy intake in individuals^(42,43)^. The point that should be considered here is that meal skipping may make adherence to a balanced diet – one of the goals of SN – more difficult, and by leading to the consumption of lower-quality, energy-dense foods, it may negatively affect health status and thus sustainability in the long term^(44)^. During pregnancy, choosing nutritious, healthy and plant-focused foods may influence hunger and satiety signals^(45)^, leading to a conscious reduction in the number of meals. If this occurs as a dietary pattern that meets nutrient requirements rather than being unhealthy or restrictive, a reduction in the number of meals – associated with conscious consumption and motivation to reduce food waste – is thought to support environmental sustainability.

In supporting SN, it is important to exhibit food purchase, reduction of food waste, preferred food type, seasonal and local food consumption^(20)^. Food waste negatively affects SN as more production is needed to feed an equal number of people^(46)^. In this study, a relationship was found between SN score and food waste reduction in pregnant women. Additionally, a positive relationship was observed between PCT and SN score in pregnant women. PCT is not always directly associated with overeating; the underlying behaviour aimed at reducing food waste may encourage not leaving food on the plate and can be an important component of SN behaviour^(47)^. In pregnant women, the tendency to finish the food on the plate may be observed with the thought of feeding the baby; this should not be considered overeating, and it may be thought to help reduce food waste. In a study evaluating the relationship between food waste concern, eating behaviour and body weight in adults, it was observed that the PCT increased in individuals who were concerned about food waste^(47)^. Therefore, the PCT in order to reduce food waste is an approach that supports sustainability, but considering that pregnancy is a special period, the content of the food consumed is also very important. Additionally, in our study, a significant relationship was found between the PCT and the consumption of seasonal and local foods in pregnant women; however, knowledge on this topic in the literature is quite limited. PCT is known to be associated with the behaviour of avoiding food waste^(47)^. Studies report that the consumption of seasonal and local foods is closely related to perceiving food as valuable and being aware of the production process, and that it can help reduce food waste^(48,49)^. This suggests that pregnant women who prefer seasonal and local foods may have higher awareness of the value of food and may be reluctant to waste it, which could in turn lead to a higher PCT. Furthermore, the perception of seasonal and local foods as beneficial for health, along with pregnant women’s increased interest in healthy foods during this period, may have reinforced their attitude not to waste these foods and increased their PCT.

Whether individuals’ preferences depend on the environmental effects of food is related to the food preference dimension^(20)^. Consumers make the food chain more sustainable with their food preferences and contribute to the prevention of chronic diseases, especially obesity, and malnutrition^(50)^. It is known that sustainable diets are plant-based; the main food groups consist of vegetables and fruits, whole grains, legumes and dairy products, and moderate consumption of nuts and fats and limited consumption of animal foods are recommended^(51)^. In this study, it was determined that food preference, one of the sub-dimensions of the behaviour scale for SN, was negatively associated with GWG. In other words, it can be said that pregnant women with a low tendency towards sustainable food preferences have higher GWG. Studies have found that the Mediterranean diet, a healthy and sustainable dietary model, prevents excessive GWG^(52–54)^. Physical activity during pregnancy reduces the risk of common pregnancy complications associated with high BMI, such as prevention of excessive GWG, gestational hypertension and diabetes, and preterm birth, especially in supporting the reduction of low back pain^(55)^. In this study, pre-pregnancy high BMI and low physical activity levels were identified as factors predicting excessive weight gain during pregnancy, and this finding is supported by the literature. In a meta-analysis evaluating the relationship between interventions based on healthy and balanced diet and physical activity during pregnancy and GWG, it was determined that diet and physical activity reduced GWG^(56)^. It is thought that physical activity and pre-pregnancy BMI should not be overlooked in preventing excessive GWG. Additionally, in our study, SN and PCT were observed not to be significant predictors of GWG, and this result may have been influenced by certain circumstances. The first of these may be the inclusion of women in the third trimester in our study; however, GWG is influenced by many physiological, hormonal, metabolic and behavioural factors that emerge from the early stages of pregnancy^(57)^. Therefore, in the later stages of pregnancy (third trimester), the impact of specific eating-related behaviours such as PCT and SN on weight gain may have diminished, and thus SN and PCT may not have significantly predicted GWG. Furthermore, it is thought that the effect of SN on GWG may not be direct but rather mediated indirectly through food preference and diet quality. This is because sustainable diets are plant-based, have low energy density and contribute to fibre intake^(58)^, all of which are closely related to diet quality (food preference). Although food preference was significantly associated with GWG, it did not predict GWG, suggesting that the effect of SN on GWG may be indirect. As previously noted, PCT does not necessarily result in overeating in every case; in the context of pregnancy, it may be related to preventing food waste and supporting adequate nutrient intake, which could have influenced the predictive effect of PCT on GWG.

### Strengths and limitations

This study has several limitations. First, dietary intake was not assessed; therefore, information on participants’ energy and nutrient intakes was not available. Second, pre-pregnancy weight was based on self-reported data, which may be subject to recall bias. Third, since the data were collected from a single state hospital in the Alanya district of Antalya, the findings may not be generalisable to all pregnant women in Türkiye. Finally, the cross-sectional design limits the ability to establish causal relationships between variables. Additionally, the internal consistency of the Turkish Plate Clearing Tendency Scale was relatively low in this sample (Cronbach’s *α* = 0·593), which may indicate limited reliability in this specific pregnant population and could have influenced the observed associations. The relatively small number of participants in the high physical activity category may have limited the precision of the estimated association. On the other hand, our study also has some strengths. In addition to the factors known to affect GWG in pregnant women such as age, education, income level and pre-pregnancy BMI, examining the relationship between PCT and sustainable eating behaviour with excessive GWG will provide a different perspective to the literature.

## Conclusion

This study, which examines PCT and SN behaviour thought to affect GWG, has revealed various findings. The findings revealed that, although there was a relationship between food preference and GWG, PCT and SN did not significantly predict GWG. The variables that significantly predicted GWG were identified as pre-pregnancy BMI and physical activity. Our study findings highlight the importance of factors such as pre-pregnancy BMI, physical activity level and dietary preferences in the management of GWG. In conclusion, it is important to develop multidimensional intervention programmes including portion control, physical activity and nutrition education to support weight control during pregnancy. Sustainable and controlled nutritional behaviour of pregnant women in a way that protects and supports their health status and is sensitive to the environment may positively affect maternal and fetal health. In the future, it is recommended that studies in which the nutrient intakes of pregnant women are also comprehensively evaluated and causal relationships are examined in a larger sample should be carried out.

## Table 1. Long description

The table presents general characteristics of participants in a study. It includes data on age groups, education status, income levels, pre-pregnancy BMI, gestational weight gain classification, physical activity levels, plate-clearing tendency score, sustainable nutrition score, meal skipping behavior, and foods consumed during snacks. The table has 18 rows and 5 columns, with headers for count, percentage, mean, and standard deviation. Key trends include the majority of participants being between 31 and 44 years old, having an income above the minimum wage, and having a normal pre-pregnancy BMI. Most participants had moderate or low physical activity levels, and the most frequently skipped meal was snacks. Yogurt, milk, fruit, and nuts were the most preferred snacks.

Navigate back to Table 1..

## Table 2. Long description

The table presents data on plate-clearing tendency and sustainable nutrition scores across various study variables. It includes mean and standard deviation for plate-clearing tendency and sustainable nutrition scores, along with P-values indicating statistical significance. The variables include age groups (19-30 years and 31-44 years), education status (primary/secondary school, high school, bachelor’s and postgraduate), income levels (below, at, and above minimum wage), pre-pregnancy BMI (less than 25 kg per meters squared and 25 kg per meters squared or more), gestational weight gain classification (insufficient, normal, and excessive), physical activity level (low, moderate, and high), and meal skipping behavior (yes, no, and sometimes). Notable trends include significant differences in plate-clearing tendency scores based on age, income, and gestational weight gain classification, and significant differences in sustainable nutrition scores based on age, education level, income, physical activity level, and meal skipping status.

Navigate back to Table 2..

## Table 3. Long description

The table presents correlation coefficients between sustainable nutrition score, its sub-dimensions, and plate-clearing tendency score with various study variables. It includes nine rows and nine columns, with row labels and column headers such as Sustainable nutrition score, Food preference, Food waste reduction, Seasonal and local food consumption, Food purchase, Plate-clearing tendency score, Age, Pre-pregnancy BMI, and Gestational weight gain. Notable correlations include significant positive relationships between the sustainable nutrition score and its sub-dimensions like food preference, food waste reduction, seasonal and local food consumption, and food purchase. Additionally, a significant positive correlation exists between the sustainable nutrition score and the plate-clearing tendency score. The plate-clearing tendency score is positively associated with seasonal and local food consumption and food purchase. Gestational weight gain shows a negative association with food preference.

Navigate back to Table 3..

## Table 4. Long description

The table presents factors predicting the likelihood of excessive gestational weight gain, analyzed through a binary logistic regression model. It includes variables such as plate-clearing tendency score, sustainable nutrition score, pre-pregnancy BMI, age, education level, income, and physical activity level. The table has seven rows and four columns, with column headers labeled Variable, OR, 95% CI for OR, and P. Each row lists a variable along with its corresponding odds ratio, confidence interval, and p-value. Notably, pre-pregnancy BMI shows a significant increase in the likelihood of excessive gestational weight gain with an odds ratio of 1.490 and a p-value of less than 0.001. High levels of physical activity significantly reduce the likelihood with an odds ratio of 0.214 and a p-value of 0.016. Other variables such as plate-clearing tendency score, sustainable nutrition score, age, education level, and income do not show significant associations with excessive gestational weight gain.

Navigate back to Table 4..
